# Construction of a ceRNA network and screening of potential biomarkers and molecular targets in male smokers with chronic obstructive pulmonary disease

**DOI:** 10.3389/fgene.2024.1376721

**Published:** 2024-06-12

**Authors:** Jihua Zhang, Shuanglan Xu, Jie Liu, Ting Liu, Zeqin Fan, Yunchun Zhou, Jorina Basnet, Liqiong Zhang, Xiao Li, Jiao Yang, Xiqian Xing

**Affiliations:** ^1^ Department of Respiratory Medicine, The People’s Hospital of Yuxi City, The Sixth Affiliated Hospital of Kunming Medical University, Yuxi, Yunnan, China; ^2^ Key Laboratory of Respiratory Disease Research of Department of Education of Yunnan Province, Department of Respiratory Medicine, The Affiliated Hospital of Yunnan University, The Second People’s Hospital of Yunnan Province, Kunming, Yunnan, China; ^3^ Department of Dermatology and Venereology, The Second Affiliated Hospital of Kunming Medical University, Kunming, Yunnan, China; ^4^ Graduate School, Kunming Medical University, Kunming, Yunnan, China; ^5^ First Department of Respiratory Medicine, The First Affiliated Hospital of Kunming Medical University, Kunming, Yunnan, China

**Keywords:** chronic obstructive pulmonary disease, circular RNAs, competitive endogenous RNA, biomarker, target

## Abstract

**Background:**

Circular RNAs (circRNAs) play an important role in the occurrence and development of diseases. However, the role of circRNAs in male smokers with chronic obstructive pulmonary disease (COPD) remains unclear.

**Methods:**

Stable COPD patients and healthy controls were recruited. Peripheral blood mononuclear cells (PBMCs) were extracted. After high-throughput RNA sequencing (RNA-Seq) of PBMCs, a bioinformatics method was used to analyse differentially expressed (DE) circRNAs (DEcircRNAs) and mRNAs (DEmRNAs).

**Results:**

Total of 114 DEcircRNAs and 58 DEmRNAs were identified. Functional enrichment analysis showed that processes related to COPD include the regulation of interleukin (IL)-18, IL-5 and the NLRP3 inflammasome; differentiation of T helper type 1 (Th1), Th2, and Th17 cells, and the AMPK, Wnt, JAK-STAT, and PI3K-Akt signalling pathways. In the protein–protein interaction (PPI) network, the core genes were MYO16, MYL4, SCN4A, NRCAM, HMCN1, MYOM2, and IQSEC3. Small-molecule prediction results revealed potential drugs for the COPD treatment. Additionally, the circRNA-miRNA-mRNA competitive endogenous RNA (ceRNA) regulatory network was constructed.

**Conclusion:**

This study identified a set of dysregulated circRNAs and mRNAs and revealed potentially important genes, pathways, new small-molecule drugs and ceRNA regulatory networks in male smokers with COPD. These circRNAs might be prospective biomarkers or potential molecular targets of the ceRNA mechanism for COPD.

## 1 Introduction

Chronic obstructive pulmonary disease (COPD), a progressive and irreversible lung disease, was the third leading cause of death globally until 2019, when it fell to the fourth leading cause of death globally due to the impact of the COVID-19 pandemic. Despite this drop in ranking, high rates of morbidity, disability, and mortality of COPD remain a global public health issue causing a very large social and economic burden worldwide (Global burden, 2024). The Global Burden of Disease Study reported a death of 3.2 million COPD patients globally in 2017 (Gbd, 2018). Much research has confirmed cigarette smoking as one of the major risk factors for COPD, and passive smoking exposure was independently associated with COPD (Hagstad et al., 2014; Wang et al., 2018a); however, the mechanism of smokers with COPD is unclear. For example, although lung fibroblasts have been identified as an important component of the remodelling process in COPD, the effect of smoking on lung fibroblasts in COPD subjects remains poorly understood (Garcia-Ryde et al., 2023). Whilst multiciliated cell loss or dysfunction is common in smokers and patients with COPD, it is not clear whether this contributes to the development of COPD lesions in the lungs of smokers (Richmond et al., 2024). In addition, the role of newly identified endogenous non-coding RNAs, which are genetically regulated in COPD, is not well understood (Shen et al., 2024). Therefore, there is of great significance to discover novel biomarkers or molecular mechanisms to promote the early screening, detection and management of smokers with COPD.

Circular RNAs (circRNAs) are newly identified non-coding RNA molecules that were originally considered to be “noise” from transcription that lacked any important biological effects (Cocquerelle et al., 1993). Subsequently, increasing evidence shows that abnormally expressed circRNAs are involved in many important pathophysiological processes and could be used as diagnostic biomarkers and therapeutic targets (Memczak et al., 2013; Sheng et al., 2018; Qiu et al., 2019; Santer et al., 2019; Sheng et al., 2020). It is worth mentioning that circRNAs act as a natural sponge to adsorb miRNAs and regulate the expression of downstream genes, which is as the definition of the mechanism of the competitive endogenous RNA (ceRNA) hypothesis (Salmena et al., 2011). This circRNA-related mechanism has received increasing attention in the pathogenesis of various human tumours, such as hepatocellular cancer, breast cancer and prostate cancer (Huang et al., 2019; Sang et al., 2019; Wang et al., 2019). To date, the significant function and ceRNA mechanism of circRNAs in the pathogenesis of COPD remains inconclusive.

In the current study, we investigated deregulated circRNAs and mRNAs from peripheral blood mononuclear cells (PBMCs) in male smokers with COPD by using high-throughput RNA sequencing (RNA-Seq) and bioinformatics analysis. This study aimed to explore possible underlying new biomarkers or molecular regulation mechanisms of circRNAs in COPD.

## 2 Materials and methods

### 2.1 Participant screening

Ten male clinical participants who smoked, including five stable COPD patients and five healthy controls, were selected on November 2019. It should be noted that the 10 smokers were in a state of abstinence when they participated in the experiment. The inclusion criteria were as follows: (1) COPD diagnosed using the ratio of postbronchodilator the forced expiratory volume in the first one second to the forced vital capacity of the lungs (FEV1/FVC). Combined with clinical manifestations, if postbronchodilator FEV1/FVC <70%, the patients were diagnosed with COPD. (2) The age of male smokers was between 50 and 80 years old, and the number of years of smoking was between 30 and 50 years. (3) The participants were from Yunnan Province to exclude the impact of different altitudes from different provinces on COPD. (4) There were no other respiratory diseases, including asthma, bronchiectasis, pulmonary fibrosis, lung tumor and tuberculosis. Healthy control individuals had no clinical symptoms of any respiratory disease, such as cough, sputum, and wheezing.

All the data were expressed as the mean ± standard deviation (SD). This study was approved by the Ethics Committee of The Affiliated Hospital of Yunnan University (No. 2020139), and each participant signed the informed consent form before the collection of blood samples.

### 2.2 Blood sample collection and PBMC extraction

Peripheral blood samples (20 mL) were collected from the median cubital vein from all participants. Then, they were placed into 5 mL purple-head EDTA-K2 anticoagulation tubes and mixed gently by inversion for subsequent experiments. Based on the manufacturer’s instructions for Human Lymphocyte Separation Medium (DAKEWEI, Beijing, China), PBMCs were isolated and extracted within 4 h.

### 2.3 High-throughput RNA-Seq

Total RNA was extracted from PBMCs using TRIzol reagent (Invitrogen, USA) according to the manufacturer’s instructions. After successful RNA quality and integrity testing, the ribosomal RNA removal kit was used to remove ribosomal RNA (rRNA) from the total RNA, and then the RNA was broken into fragments of approximately 300 bp in length by ion interruption. cDNA libraries were generated by polymerase chain reaction (PCR) amplification for the enrich library fragments. Then, the Agilent 2,100 Bioanalyzer was used for quality inspection of the library, including the total concentration and effective concentration. After the quality inspection results were passed, next-generation sequencing (NGS) technology was used to perform paired-end sequencing of these libraries based on the Illumina HiSeq sequencing platform (Personalbio, Shanghai, China).

After data filtering (Cutadapt) and quality evaluation (FastQC), the RNA-Seq data were compared to the reference genome (TopHat2), and the data source for the reference genome was the Ensembl database (http://www.ensembl.org/, Homo_sapiens.GRCh38.dna.primary assembly.fa). Then, we used HTSeq to compare the read count value of each gene as the original expression of the gene and Fragments Per Kilobase of transcript per Million mapped reads (FPKM) to normalize the expression. These RNA-Seq data include circRNAs and mRNAs.

### 2.4 Differential expression analysis

After filtering the original offline data, the sequences from these data were compared to that of the reference genome of humans, and the expression of each gene was calculated. R software (version number: 3.4.1, http://www.r-project.org/) was used to normalize and standardize the data, and the DESeq R package was used for differentially expressed gene (DEG) analysis (Sun et al., 2013). Under the conditions of a |log2FoldChange| value > 1 and a significant *p*-value < 0.05, differentially expressed (DE) circRNAs (DEcircRNAs) and mRNAs (DEmRNAs) were identified. To show the characteristics of DEGs, volcano maps and hierarchical bidirectional clustering analysis were generated using the ggplots2 R package. In addition, we used the circlize R package to label DEGs in the genome in a genomic circle diagram based on the genomic information and the RNA differential expression analysis results.

### 2.5 Functional and pathway enrichment analyses

Gene Ontology (GO, http://www.geneontology.org/) functional enrichment analysis of DEGs covers three domains, i.e., biological processes (BPs), cellular components (CCs), and molecular functions (MFs). The Kyoto Encyclopedia of Genes and Genomes (KEGG, http://www.kegg.jp/) contains a gene information database, a system information database, and a chemical information database. The topGO and pathview R packages were used for GO enrichment analysis, and a *p*-value <0.05 was defined as statistically significant.

### 2.6 PPI network

The Search Tool for the Retrieval of Interacting Genes/Proteins (STRING) database (https://string-db.org/) was used to construct the protein–protein interaction (PPI) networks of integrated DEGs (Szklarczyk et al., 2019). The minimum required interaction score was 0.15. The obtained results were imported into Cytoscape version 3.6.1. The cytoHubba plugin provides a user-friendly interface to explore important nodes in PPI networks by the multiscale curvature classification (MCC) algorithm (score ≥5), and it was used to select the hub node, which is named the core gene or core protein (Chin et al., 2014).

### 2.7 Identification of small-molecule candidate drugs

The Connectivity Map (CMap), a database of biological applications, revealed the relationship of small-molecule drugs, gene expression levels and interrelated diseases (Gao et al., 2019). In other words, the gene expression profile was used to establish the association between genes, diseases and drugs, which should help scholars to quickly identify highly associated genes with a disease, identify the main chemical structure of a molecule, and summarize the possible directions of the mechanism of drug molecules.

### 2.8 Construction of the ceRNA regulatory network

The regulatory relationships of the DEcircRNA-DEmiRNA pairs and DEmiRNA-DEmRNA pairs were predicted based on the CircInteractome (https://circinteractome.nia.nih.gov/), StarBase (http://starbase.sysu.edu.cn/), TargetScan (http://www.targetscan.org/), miRTarBase (http://mirtarbase.mbc.nctu.edu.tw/), miRDB (http://www.mirdb.org/), miRanda (http://www.microrna.org/) and miRBase (http://www.mirbase.org/) databases (Fan et al., 2016; Fan et al., 2018). Then, the intersection of predicted target genes and DEmRNAs was analysed by the custom Venn diagram tool. The circRNA acts as a competitive endogenous RNA (ceRNA) to competitively bind with miRNA and to regulate the expression of downstream genes and proteins. According to the ceRNA theory, a circRNA-miRNA-mRNA ceRNA regulatory network was constructed by Cytoscape version 3.6.1 (Shannon et al., 2003), integrating circRNA-miRNA pairs and miRNA-mRNA pairs.

### 2.9 Real-time quantitative PCR (RT-qPCR)

Total RNA was extract from PBMCs by TRIzol (Invitrogen, USA). cDNA was synthesized using a reverse transcription kit (Vazyme Biotech Co., Ltd., Nanjing, China). qPCR was conducted using the SYBR Master Mix Kit (Vazyme, Nanjing, China) in an ABI 7500 PCR System (Applied Biosystems, CA, USA). The PCR conditions were 95°C for 5 min, followed by 40 cycles of 95°C for 15 s and 60°C for 30 s. Data were normalized using small nuclear RNA 6 (U6) for circRNAs, GAPDH for mRNAs as a reference gene. The relative expression was calculated using the 2^−ΔΔCT^ method. The primer for RT-qPCR in Table 1.

**TABLE 1:** Primer for Quantitative real-time PCR (qRT-PCR).

Gene names	Forward-primer sequence (5′-3′)	Reverse-primer sequence (5′-3′)
hsa_circ_006155	GCC​AGG​ACT​CCC​AAT​CTT​GTA​A	AGT​TAC​TGG​TGG​AGT​TGA​ACC​TT
hsa_circ_007446	TTC​TCA​AGT​GGT​TCA​GGT​GGT​T	CAC​TGC​TTC​CTC​CAC​ATT​CCT​C
hsa_circ_019015	CTG​ACC​ATG​TAA​AGC​AAT​GCC​A	GGC​CTT​CTC​ATC​TTG​CTT​TGA​G
hsa_circ_031080	TTC​CTG​ACA​ATG​GCA​GAA​TGT​CAA​T	ACC​ACC​AAA​GGT​GTG​AAA​GAA​C
hsa_circ_038146	CAA​ATT​CTG​GAG​CCT​GCA​TTG​A	AAT​TCA​ACA​TCT​CAG​CTG​TGC​C
hsa_circ_041434	CAA​CCT​TGG​AAA​CAT​TGC​TCA​GA	GCT​GCA​TGG​TCT​GCT​AAC​ATT
U6	CTCGCTTCGGCAGCACA	AAC​GCT​TCA​CGA​ATT​TGC​GT
HYDIN	TTA​GAG​GCT​GTG​TCA​TTG​GAC​C	AGT​CCA​CAT​GCT​GCT​CAC​AAT​A
MUC19	CAA​CAC​CAT​CTC​ACC​TAG​CAG​T	TTA​CTT​CCA​GCC​TCA​GTG​GTT​C
MYZAP	TTA​ACA​GAA​ACC​CAG​GCC​AAG​A	GGC​ATC​ACA​ATT​TCA​CGA​GTC​C
NRCAM	TAG​ACT​GTG​CCT​TCT​TTG​GGT​C	AAC​TTC​ATT​CTT​CGC​CAT​CCC​T
RNF17	TGG​AAG​GAG​GGT​ATC​CAG​ATC​A	AGC​TGT​AGC​AAC​CTC​ACA​ATC​A
SHE	AGC​TAT​ACG​ACA​CTC​CCT​ACG​A	CTC​AGC​TCC​TTC​AAA​CTG​GAC​T
GAPDH	GCAAGTTCAACGGCACAG	GCC​AGT​AGA​CTC​CAC​GAC​AT

### 2.10 Statistical analysis

The statistical analyses were performed using R software and SPSS. Among them, R software was used for the visualisation of the results of GO enrichment, KEGG function and differential gene analysis, and t-tests for RT-qPCR were performed with SPSS version 24.0 (SPSS, IL, USA). Additionally, independent samples t-tests were carried out using SPSS for age and years of smoking, and *p* > 0.05 was deemed to be not statistically significant.

## 3 Results

### 3.1 Participant characteristics

The characteristics of the ten eligible participants are presented in Table 2, including five COPD patients and five healthy controls. All participants were male smokers with good general physical fitness from Yunnan Province. In the COPD group, the age range was 53–76 years, the pack-years of smoking ranged from 40 to 52 years, and the FEV1/FVC values were all <70%. In the control group, the age range was 58–76 years, the pack-years of smoking ranged from 30 to 48 years, and the FEV1/FVC values were all >70%. There were no statistically significant differences in age (66.00 ± 8.60 years vs. 65.40 ± 6.73 years, *p* > 0.05) or number of years of smoking (44.00 ± 4.95 years vs. 38.00 ± 8.03 years, *p* > 0.05) between the two groups.

**TABLE 2:** Characteristics of the ten eligible participants.

Rank	Gender	Age (y)	Years of smoking (y)	Previous disease history	FEV1/FVC (%)
COPD 1	Males	63	43	hypertension, hyperlipidemia	69.90
COPD 2	Males	76	52	none	65.61
COPD 3	Males	69	40	none	59.89
COPD 4	Males	69	45	hyperlipidemia	58.49
COPD 5	Males	53	40	appendectomy	28.99
Control 1	Males	76	48	none	85.89
Control 2	Males	63	45	none	73.93
Control 3	Males	63	35	benign prostatic hyperplasia	74.52
Control 4	Males	67	30	none	82.65
Control 5	Males	58	32	none	79.44

Abbreviations: y, years; FEV1/FVC, ratio of forced expiratory volume to forced vital capacity in the first second.

### 3.2 Examination of the sample quality

**FIGURE 1:**
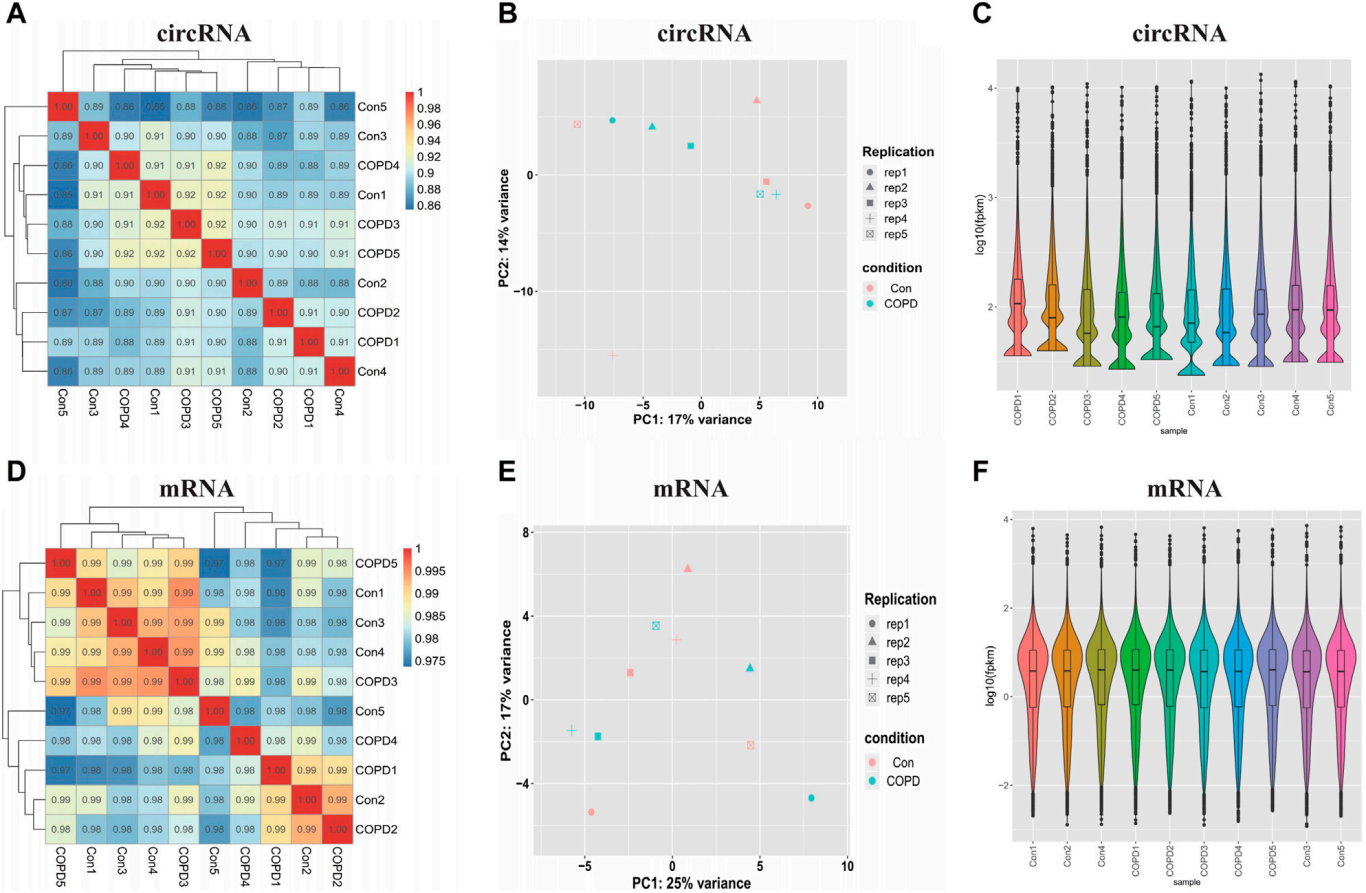
Examination of samples quality for circRNAs and mRNAs. **(A)** The Correlation analysis of circRNAs samples. **(B)** The Principal Components Analysis of circRNAs samples. **(C)** The violin plots show the distribution of circRNAs. **(D)** The Correlation analysis of mRNAs samples. **(E)** The Principal Components Analysis of mRNAs samples. **(F)** The violin plots show the distribution of mRNAs. Con, Control Group; COPD, Chronic obstructive pulmonary disease.

Correlation analysis suggested that the correlation coefficient was highly strong (between 0.8-1), indicating that the similarity of the expression patterns between samples was high (Figures 1A, D). Principal component analysis (PCA) determined patterns between similar samples, and if the distance is closer, the similarity between the samples is higher. Our results showed good similarity between samples in Figures 1B, E. In addition, the distributions of the datasets for the circRNAs/mRNAs of the ten samples were nearly identical after normalization, as shown in violin plots (Figures 1C, F).

### 3.3 Computational analysis of DEcircRNAs and DEmRNAs

**FIGURE 2:**
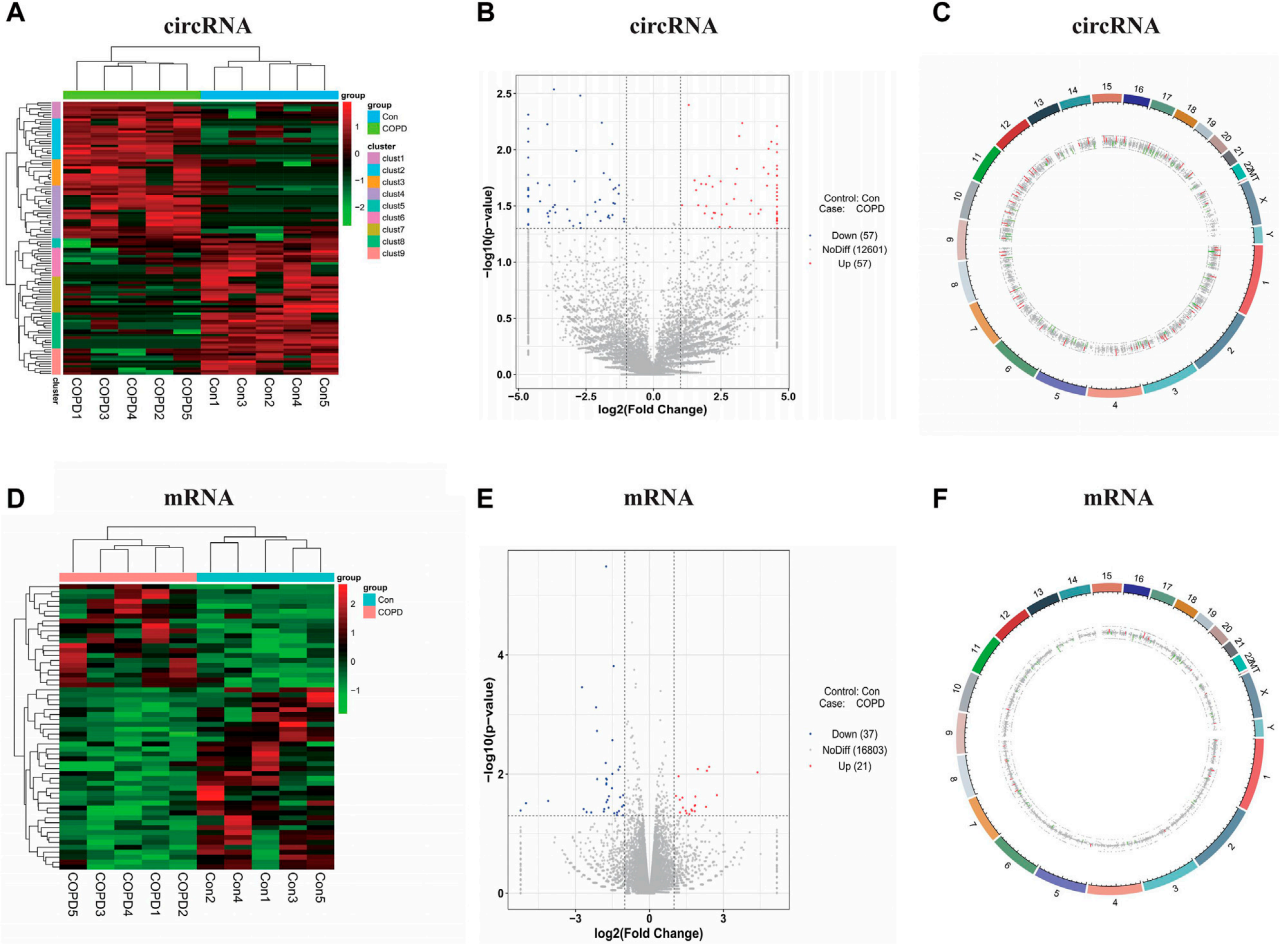
Identification of the significant expression changes of circRNAs and mRNAs. **(A)** The heatmaps of DEcircRNAs. **(B)** The volcano plots of DEcircRNAs. **(C)** The genomic circle diagram of DEcircRNAs. **(D)** The heatmaps of DEmRNAs. **(E)** The volcano plots of DEmRNAs. **(F)** The genomic circle diagram of DEmRNAs. The red, blue/green and black dots represent genes that are upregulated, downregulated and not significantly differentially expressed in the heatmaps or volcano plots. Con, Control Group; COPD, Chronic obstructive pulmonary disease.

By comparing the COPD group with the healthy control group using the screening criteria, there were 114 significant DEcircRNAs, in which 57 were upregulated (including the top 10 genes: hsa_circ_032857, hsa_circ_019208, hsa_circ_024906, hsa_circ_035125, hsa_circ_010451, hsa_circ_019753, hsa_circ_031856, hsa_circ_040189, hsa_circ_021622, and hsa_circ_003470), and 57 were downregulated (including the top 10 genes: hsa_circ_007446, hsa_circ_016302, hsa_circ_019528, hsa_circ_019015, hsa_circ_038146, hsa_circ_026711, hsa_circ_026970, hsa_circ_001331, hsa_circ_012036, and hsa_circ_012997), and 58 significant DEmRNAs, in which 21 were upregulated (including the top 10 genes: KCNJ12, SHE, GCOM1, NRCAM, MYZAP, TMEM178B, SEMA5A, VSTM4, MYOM2, and MYL4), and 37 were downregulated (including the top 10 genes: GDF15, RNF17, HYDIN, HLA-DQA1, RSPO4, MUC19, TCF7L1, COL26A1, HOXA10, and C7orf61) that were identified (Supplementary Table S1). DEcircRNAs and DEmRNAs were confirmed by a heatmap (Figures 2A, D) and volcano plot, respectively (Figures 2B, E). In addition, we marked DEGs on the genomic circle diagram to display the position of the DEGs on the chromosome in detail (Figures 2C, F).

### 3.4 Functional and pathway enrichment analyses

**FIGURE 3:**
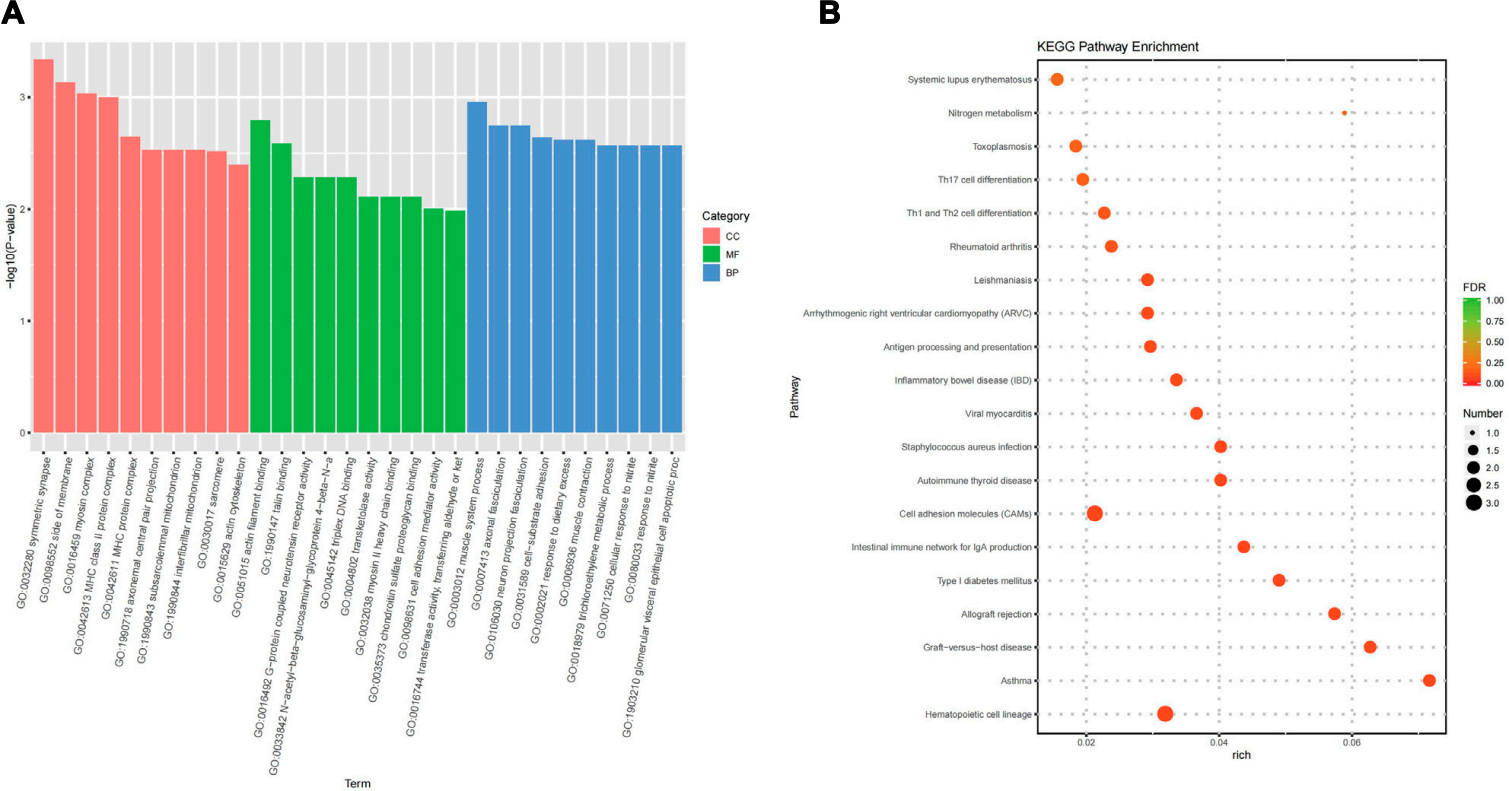
Functional and pathway enrichment analysis. **(A)** Top 10 clusters GO Functional enrichment analysis of DEmRNAs. **(B)** Top 20 clusters KEGG pathway enrichment analysis of DEmRNAs.

GO functional enrichment analysis showed that the main pathways enriched for biological process primarily were the response to resveratrol, response to metformin, positive regulation of interleukin (IL)-18 production, positive regulation of IL-5 secretion, multicellular organismal signalling, positive regulation of NLRP3 inflammasome complex assembly, positive regulation of endothelial cell chemotaxis, response to nitrite, cellular response to nitrite, cell-substrate adhesion, and axonal fasciculation. The cellular component included the cytoplasmic side of the plasma membrane, cell surface, axonal surface, symmetric synapse, side of the membrane, and myosin complex, major histocompatibility complex (MHC) class II protein complex, and MHC protein complex. The MFs included IL-2 binding, talin binding, protein-coupled neurotensin receptor activity, triplex DNA binding, transketolase, transferase activity, and transferring aldehyde or ketones groups Figure 3A shows the top 10 clusters of GO functional enrichment analysis of DEmRNAs.

KEGG pathway enrichment analysis found that the signalling pathways related to COPD included the differentiation of Th1, Th2 and Th17 cells, apelin signalling pathway, AMPK signalling pathway, Wnt signalling pathway, JAK-STAT signalling pathway, and PI3K-Akt signalling pathway. Figure 3B shows the top 20 clusters of KEGG pathway enrichment analysis of DEmRNAs.

### 3.5 PPI network

**FIGURE 4:**
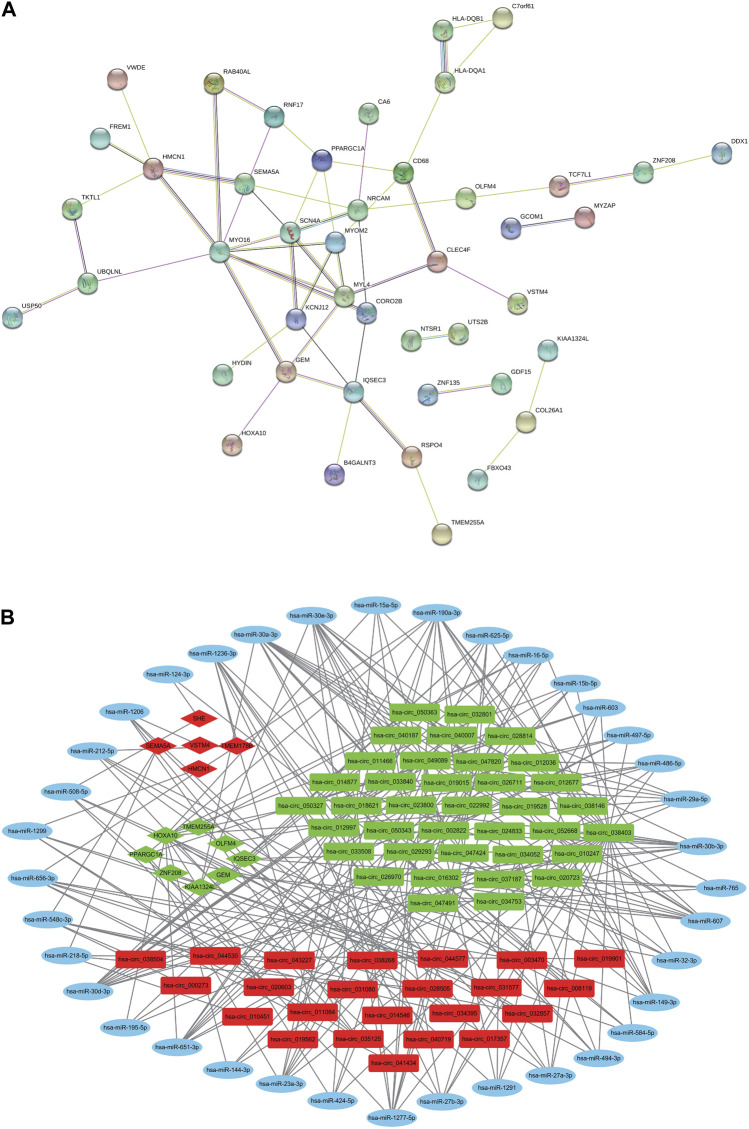
PPI network and circRNA-mediated ceRNA Regulatory Network. **(A)** PPI network analysis of DE mRNAs. **(B)** The construction of the circRNA-miRNA-mRNA ceRNA regulatory network. Red nodes indicate upregulated RNAs, green nodes indicate downregulated RNAs, and blue nodes indicate none detected miRNAs. Circles indicate DEmiRNAs, rectangles indicate DEcircRNAs, and diamonds indicate DEmRNAs.

As shown in Figure 4A, the DEmRNAs are plotted as a PPI network showing the interaction between multiple proteins. In the PPI map, the core genes in the network are MYO16, MYL4, SCN4A, NRCAM, HMCN1, MYOM2, and IQSEC3.

### 3.6 Prediction of small-molecule drugs

Based on DEGs between the COPD patients and healthy controls, the CMap database was utilized to screen candidate small-molecule drugs. Suitable parameters included a significant *p*-value < 0.05 and an absolute enrichment value > 0.80. As Table 3 shows, there were negative correlations between hycanthone, ascorbic acid, canavanine, puromycin and lincomycin; therefore, they were considered to be potential drugs for the treatment of COPD.

**TABLE 3:** Results of the connectivity map analysis.

Rank	CMap name	Mean	N	Enrichment	*p*-value	Specificity	Percent
1	sulfinpyrazone	0.325	4	0.816	0.00211	0	50
2	hycanthone	−0.649	4	−0.882	0.00048	0	100
3	ascorbic acid	−0.587	4	−0.868	0.0006	0	100
4	canavanine	−0.602	3	−0.855	0.00603	0.0103	100
5	puromycin	−0.433	4	−0.814	0.00223	0.0448	75
6	lincomycin	−0.517	3	−0.806	0.01448	0.0108	66

### 3.7 Construction of the circRNA-mediated ceRNA regulatory network

The ceRNA regulatory network of circRNAs-miRNAs-mRNAs (Figure 4B) contains 60 DEcircRNAs (23 upregulated, 37 downregulated), 39 predicted DE miRNAs (DEmiRNAs; 28 upregulated, 11 downregulated) and 13 DEmRNAs (5 upregulated, 8 downregulated). There were 225 ceRNA pairs in the network. As shown in Supplementary Table S2, the negative regulatory relationships among the DEcircRNAs, DEmiRNAs, and DEmRNAs in the ceRNA network are listed in detail.

### 3.8 Validation of differentially expressed genes

**FIGURE 5:**
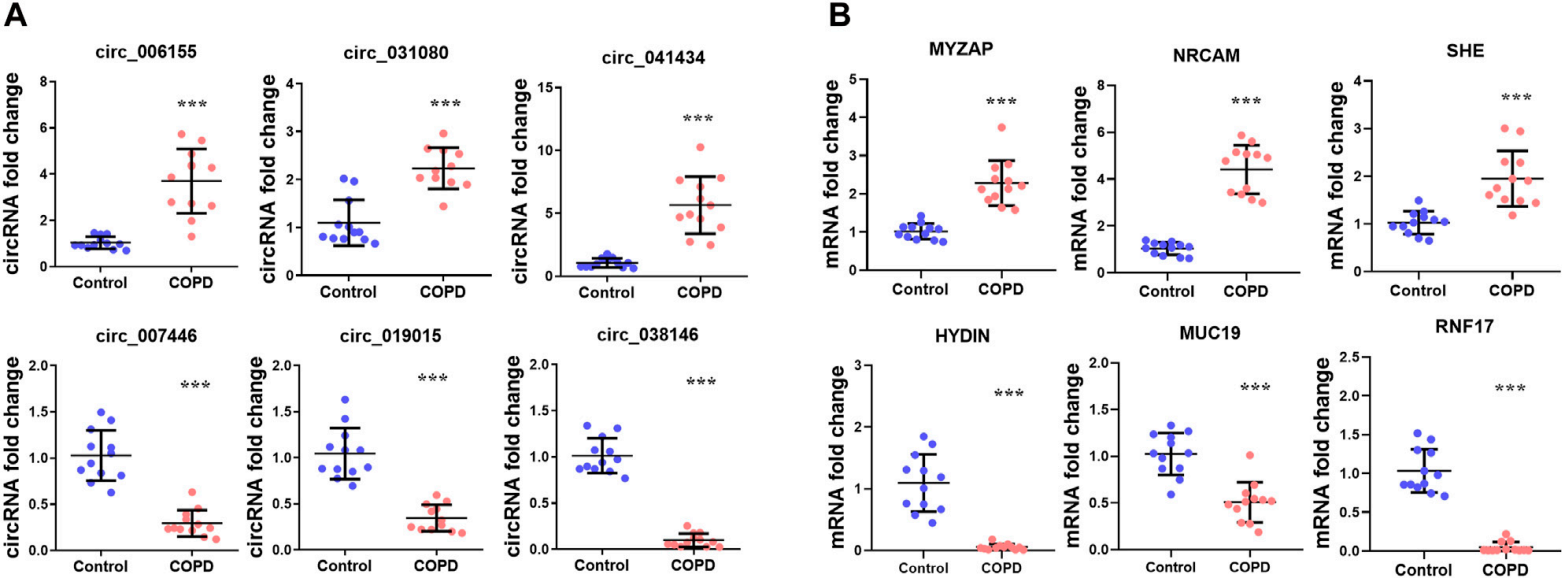
The expression of RNAs in the COPD group compared with the normal control group. **(A)** Expression of three upregulated and three downregulated circRNAs. **(B)** Expression of three upregulated and three downregulated mRNAs.

To verify the RNA sequencing data, we selected six identified circRNAs for the analysis of RT-qPCR including three upregulated circRNAs and three downregulated circRNAs, and six candidate mRNAs including three upregulated mRNAs and three downregulated mRNAs. The results showed that hsa_circ_006155, hsa_circ_031080, hsa_circ_041434, MYZAP, NRCAM and SHE were increased, whereas hsa_circ_007446, hsa_circ_019015, hsa_circ_038146, HYDIN, MUC19, and RNF17 were decreased in COPD patients versus normal control samples. The results were consistent with the RNA-Seq data (Figure 5).

## 4 Discussion

COPD is a slowly irreversible and progressive disease of the lungs, and it is a major cause of morbidity and mortality worldwide. Long-term cigarette smoke exposure is the most important risk factor in the development of COPD. Therefore, in our study, we identified the expression patterns of circRNAs and mRNAs in PBMCs of long-term male smokers based on high-throughput RNA-Seq technology. By comparing circRNAs and mRNAs in the COPD and healthy control groups, a set of DEcircRNAs and DEmRNAs were identified in the COPD group. Subsequently, through a series of bioinformatics methods, a ceRNA network was constructed, and potential biomarkers and molecular targets were screened. To the best of our knowledge, transcriptome analysis of circRNAs and mRNAs in male smokers with COPD was performed in this study for the first time. This analysis was valuable to provide a reliable theoretical basis for COPD researchers.

With the rapid development of NGS and bioinformatics methods, circRNAs have been shown to be widespread in different species, where they are highly conserved, tissue-specific, and timing-specific molecules that have many important biological functions (Memczak et al., 2013). In a recent review, Lei and his team provided a comprehensive summary of the expression and/or function of various circRNAs related to multiple types of tumours, including the digestion, respiration, and urinary system tumours, and concluded that circRNAs may be used as helpful biomarkers or therapeutic targets in human tumours and have potential clinical application value (Lei et al., 2019). However, research on the functions and mechanisms of circRNAs in COPD is still in its infancy. The latest research identified the expression of circRNAs in PBMCs from patients with COPD by bioinformatic analysis, and validated the microarray data by RT-qPCR (Duan et al., 2020). Further, by sequencing the exosomal RNA and single-cell transcriptome of PBMC in COPD, Pei et al.'s study characterized numerous potential molecular aspects of the disease and suggested a probable link between compromised immune function and the advancement of COPD (Pei et al., 2022). Another study showed that cigarette smoke extract alters the genome-wide profiles of circRNAs in primary human small airway epithelial cells, suggesting that circRNAs might be involved in the development of COPD (Zeng et al., 2019). In contrast, our research was based on PBMCs from long-term smoking participants, and 114 significantly DEcircRNAs were screened, of which 57 DEcircRNAs were overexpressed, and 57 DEcircRNAs were underexpressed in the COPD group in comparison to the those in the control group, further indicating that DEcircRNAs may be used as biomarkers in male smokers with COPD.

GO functional enrichment analysis revealed that important functions related to COPD were the response to resveratrol and metformin and the regulation of IL-18, IL-5 and NLRP3 inflammasomes. As summarized from previous studies, the beneficial effects of resveratrol on anti-inflammatory and antioxidant activities have been confirmed in relation to many diseases, including ageing, obesity, diabetes and COPD (Liu et al., 2016; Beijers et al., 2018). Metformin is often used in the clinical treatment of diabetes. Interestingly, studies have shown that the use of metformin in diabetic patients can reduce not only the risk of COPD but also the adverse prognostic outcome of COPD (Ho et al., 2019; Tseng, 2019). It is well known that the occurrence and severity of COPD is related to abnormal airway inflammation. Activated inflammatory cells can produce a variety of the proinflammatory cytokines ILs, including IL-1 and IL-6 (Dima et al., 2015), and the multiprotein complex NLRP3 inflammasome can produce IL-1β and IL-18, which may contribute to the development of airway inflammation in patients with COPD (Wang et al., 2018b).

KEGG pathway enrichment analysis revealed that the signalling pathways related to COPD included the differentiation of Th1, Th2 and Th17 cells, AMPK signalling pathway, Wnt signalling pathway, JAK-STAT signalling pathway, and PI3K-Akt signalling pathway. These pathways have been reported in previous studies by mediating different molecular signalling pathways, regulating the expression of related genes, and then participating in the occurrence and development of COPD. In particular, these pathways mediate the inflammatory process of COPD by regulating the release and expression of inflammation-related cytokines and mediators (Heijink et al., 2013; Cui et al., 2018; Wei and Sheng Li, 2018; Zhang et al., 2018; Yu et al., 2020; Zhao et al., 2020). A study found that autophagy was significantly enhanced in locomotor muscles of stable patients with COPD by activation of the AMPK signalling pathway (Guo et al., 2013). Additionally, in our study, the core genes were MYO16, MYL4, SCN4A, NRCAM, HMCN1, MYOM2, and IQSEC3 in the PPI network, but none of these genes have been reported in COPD. Hycanthone, ascorbic acid, canavanine, puromycin and lincomycin are considered to be potential drugs for the treatment of COPD in the current study. Ascorbic acid is also known as vitamin C, which can not only improve the nutritional and antioxidant status of COPD (Pirabbasi et al., 2016) but also play a preventive role (Park et al., 2016). More research is needed to determine the functions of these genes and drugs.

In previous literature, the circRNA-related ceRNA mechanism in COPD has rarely been studied. Using cigarette smoke extraction or cigarette smoke to construct COPD cell models and mouse models, a previous study verified the ceRNA mechanism of circ0061052 *in vivo* and *in vitro* and concluded that circ0061052 was involved in cigarette smoke-induced epithelial-mesenchymal transition and airway remodelling in COPD by regulating miR-515-5p through a FoxC1/Snail regulatory axis (Ma et al., 2020). In our study, the circRNA-miRNA-mRNA ceRNA regulatory network contained 60 DEcircRNAs (23 upregulated, 37 downregulated), 39 predicted DEmiRNAs (28 upregulated, 11 downregulated) and 13 DEmRNAs (5 upregulated, 8 downregulated). There were 225 pairs of ceRNA regulatory relationships. Not surprisingly, multiple ceRNA regulatory relationship pairs related to the AMPK signalling pathway were screened, and the target gene was PPARGC1A, constituting 35 pairs of circRNA-miRNA-PPARGC1A (Supplementary Table S2). The AMPK-PPARGC1A pathway is involved in an important biological process, autophagy, which has been previously confirmed (Yang et al., 2014). Whether the screened circRNA-related genes enriched in the AMPK pathway can mediate the AMPK-PPARGC1A pathway in COPD or whether other ceRNA pairs have corresponding functions in COPD, further studies are needed to determine its complex mechanism. This provides a viable foundation for more in-depth mechanistic research in the future.

There were some limitations in our study. First, the sample size is relatively small. The conclusions of this study require further verification in larger and more diverse cohorts. Second, the regulatory networks or mechanisms analysed in this study identified only the DEcircRNAs and DEmRNAs based on bioinformatics predictions and lacks *in vitro* and *in vivo* experiments to verify these results. More comprehensive analyses and further in-depth studies with *in vitro* and *in vivo* experiments and circRNA knockdown or overexpression experiments are still needed.

## 5 Conclusion

In conclusion, this study identified a set of DEcircRNAs and DEmRNAs and revealed potentially important genes, pathways, new small-molecule drugs and a ceRNA regulatory network in male smokers with COPD. These circRNAs might become prospective diagnostic biomarkers and therapeutic targets in the clinic or provide potential AMPK-PPARGC1A-mediated autophagy ceRNA mechanism for COPD.

## Data Availability

The data presented in the study are deposited in the GEO repository, accession number GSE268499.

## Supplementary material

SUPPLEMENTARY TABLE S1 Differentially expressed circRNAs and mRNAs.

SUPPLEMENTARY TABLE S2 Relationship between negative regulation of circRNA, miRNA, and mRNA in the ceRNA network.
