# Supplementary material for: Construction of a ceRNA network and screening of potential biomarkers and molecular targets in male smokers with chronic obstructive pulmonary disease
Source: Front Genet. 2024 Jun 12;15:1376721. doi: 10.3389/fgene.2024.1376721 (PMC11199688; doi:10.3389/fgene.2024.1376721)
Supplement: Supplementary file 3 [file Table1.DOCX]

***Supplementary Material***

# **Supplementary Tables**

## **Supplementary Table 1**

**Table 1. Differentially expressed circRNAs and mRNAs.**

| **Gene symbol** | **Category** | **Regulation** | **Log_2_ Fold Change** | **P-value** |
| --- | --- | --- | --- | --- |
| hsacirc_032857 | circRNA | up | +∞ | 0.0061 |
| hsacirc_019208 | circRNA | up | +∞ | 0.0089 |
| hsacirc_024906 | circRNA | up | +∞ | 0.0105 |
| hsacirc_035125 | circRNA | up | +∞ | 0.0149 |
| hsacirc_010451 | circRNA | up | +∞ | 0.0169 |
| hsacirc_019753 | circRNA | up | +∞ | 0.0170 |
| hsacirc_031856 | circRNA | up | +∞ | 0.0220 |
| hsacirc_040189 | circRNA | up | +∞ | 0.0239 |
| hsacirc_021622 | circRNA | up | +∞ | 0.0258 |
| hsacirc_003470 | circRNA | up | +∞ | 0.0278 |
| hsacirc_038504 | circRNA | up | +∞ | 0.0320 |
| hsacirc_038268 | circRNA | up | +∞ | 0.0357 |
| hsacirc_008119 | circRNA | up | +∞ | 0.0369 |
| hsacirc_019901 | circRNA | up | +∞ | 0.0374 |
| hsacirc_002792 | circRNA | up | +∞ | 0.0405 |
| hsacirc_020825 | circRNA | up | +∞ | 0.0410 |
| hsacirc_031764 | circRNA | up | +∞ | 0.0423 |
| hsacirc_048869 | circRNA | up | +∞ | 0.0423 |
| hsacirc_028505 | circRNA | up | +∞ | 0.0429 |
| hsacirc_046533 | circRNA | up | +∞ | 0.0430 |
| hsacirc_011071 | circRNA | up | +∞ | 0.0454 |
| hsacirc_034395 | circRNA | up | +∞ | 0.0500 |
| hsacirc_041434 | circRNA | up | 4.5749 | 0.0139 |
| hsacirc_006155 | circRNA | up | 4.5742 | 0.0206 |
| hsacirc_031080 | circRNA | up | 4.3697 | 0.0085 |
| hsacirc_033543 | circRNA | up | 4.2631 | 0.0098 |
| hsacirc_031577 | circRNA | up | 4.2541 | 0.0163 |
| hsacirc_011084 | circRNA | up | 4.2073 | 0.0209 |
| hsacirc_046974 | circRNA | up | 4.0841 | 0.0144 |
| hsacirc_038837 | circRNA | up | 3.9713 | 0.0341 |
| hsacirc_002683 | circRNA | up | 3.8899 | 0.0371 |
| hsacirc_044530 | circRNA | up | 3.6245 | 0.0283 |
| hsacirc_035453 | circRNA | up | 3.5807 | 0.0373 |
| hsacirc_017357 | circRNA | up | 3.2892 | 0.0058 |
| hsacirc_018338 | circRNA | up | 3.1747 | 0.0076 |
| hsacirc_014546 | circRNA | up | 3.0898 | 0.0148 |
| hsacirc_009980 | circRNA | up | 3.0336 | 0.0202 |
| hsacirc_020603 | circRNA | up | 2.9852 | 0.0274 |
| hsacirc_019464 | circRNA | up | 2.8331 | 0.0488 |
| hsacirc_038940 | circRNA | up | 2.7116 | 0.0305 |
| hsacirc_000273 | circRNA | up | 2.5289 | 0.0377 |
| hsacirc_044577 | circRNA | up | 2.4899 | 0.0191 |
| hsacirc_000023 | circRNA | up | 2.4485 | 0.0488 |
| hsacirc_021930 | circRNA | up | 2.2803 | 0.0365 |
| hsacirc_004538 | circRNA | up | 2.2318 | 0.0363 |
| hsacirc_029082 | circRNA | up | 2.1884 | 0.0214 |
| hsacirc_012179 | circRNA | up | 2.1721 | 0.0407 |
| hsacirc_048217 | circRNA | up | 2.0674 | 0.0171 |
| hsacirc_040719 | circRNA | up | 1.9564 | 0.0318 |
| hsacirc_043227 | circRNA | up | 1.9451 | 0.0203 |
| hsacirc_023298 | circRNA | up | 1.8986 | 0.0369 |
| hsacirc_038134 | circRNA | up | 1.7697 | 0.0201 |
| hsacirc_033987 | circRNA | up | 1.6573 | 0.0312 |
| hsacirc_042602 | circRNA | up | 1.5794 | 0.0229 |
| hsacirc_040119 | circRNA | up | 1.5224 | 0.0187 |
| hsacirc_022353 | circRNA | up | 1.3067 | 0.0040 |
| hsacirc_019562 | circRNA | up | 1.0576 | 0.0312 |
| hsacirc_011466 | circRNA | down | -∞ | 0.0049 |
| hsacirc_032801 | circRNA | down | -∞ | 0.0085 |
| hsacirc_038403 | circRNA | down | -∞ | 0.0118 |
| hsacirc_052786 | circRNA | down | -∞ | 0.0167 |
| hsacirc_033107 | circRNA | down | -∞ | 0.0219 |
| hsacirc_002855 | circRNA | down | -∞ | 0.0227 |
| hsacirc_047424 | circRNA | down | -∞ | 0.0228 |
| hsacirc_024859 | circRNA | down | -∞ | 0.0258 |
| hsacirc_022245 | circRNA | down | -∞ | 0.0262 |
| hsacirc_035595 | circRNA | down | -∞ | 0.0338 |
| hsacirc_029293 | circRNA | down | -∞ | 0.0342 |
| hsacirc_047112 | circRNA | down | -∞ | 0.0346 |
| hsacirc_049089 | circRNA | down | -∞ | 0.0357 |
| hsacirc_014877 | circRNA | down | -∞ | 0.0458 |
| hsacirc_047820 | circRNA | down | -∞ | 0.0467 |
| hsacirc_007446 | circRNA | down | -4.6411 | 0.0065 |
| hsacirc_016302 | circRNA | down | -4.2875 | 0.0198 |
| hsacirc_019528 | circRNA | down | -4.2031 | 0.0288 |
| hsacirc_019015 | circRNA | down | -3.9313 | 0.0059 |
| hsacirc_038146 | circRNA | down | -3.9261 | 0.0367 |
| hsacirc_026711 | circRNA | down | -3.8985 | 0.0392 |
| hsacirc_026970 | circRNA | down | -3.8714 | 0.0445 |
| hsacirc_001331 | circRNA | down | -3.8567 | 0.0206 |
| hsacirc_012036 | circRNA | down | -3.8385 | 0.0351 |
| hsacirc_012997 | circRNA | down | -3.7204 | 0.0324 |
| hsacirc_024833 | circRNA | down | -3.6884 | 0.0029 |
| hsacirc_018621 | circRNA | down | -3.6560 | 0.0310 |
| hsacirc_023800 | circRNA | down | -3.6341 | 0.0361 |
| hsacirc_002822 | circRNA | down | -3.1951 | 0.0339 |
| hsacirc_006365 | circRNA | down | -3.1083 | 0.0429 |
| hsacirc_034753 | circRNA | down | -2.9721 | 0.0476 |
| hsacirc_022992 | circRNA | down | -2.9232 | 0.0191 |
| hsacirc_002620 | circRNA | down | -2.8577 | 0.0102 |
| hsacirc_050343 | circRNA | down | -2.7218 | 0.0450 |
| hsacirc_050327 | circRNA | down | -2.7127 | 0.0033 |
| hsacirc_033840 | circRNA | down | -2.6923 | 0.0497 |
| hsacirc_050363 | circRNA | down | -2.4460 | 0.0380 |
| hsacirc_012103 | circRNA | down | -2.1608 | 0.0293 |
| hsacirc_033508 | circRNA | down | -2.1506 | 0.0356 |
| hsacirc_044958 | circRNA | down | -1.9866 | 0.0279 |
| hsacirc_040187 | circRNA | down | -1.9623 | 0.0431 |
| hsacirc_000388 | circRNA | down | -1.9146 | 0.0058 |
| hsacirc_037187 | circRNA | down | -1.8431 | 0.0162 |
| hsacirc_045514 | circRNA | down | -1.6894 | 0.0399 |
| hsacirc_006969 | circRNA | down | -1.6628 | 0.0386 |
| hsacirc_028814 | circRNA | down | -1.6304 | 0.0405 |
| hsacirc_012677 | circRNA | down | -1.6152 | 0.0184 |
| hsacirc_043020 | circRNA | down | -1.5224 | 0.0089 |
| hsacirc_020723 | circRNA | down | -1.5186 | 0.0381 |
| hsacirc_007772 | circRNA | down | -1.4878 | 0.0298 |
| hsacirc_047491 | circRNA | down | -1.4814 | 0.0225 |
| hsacirc_040007 | circRNA | down | -1.4328 | 0.0302 |
| hsacirc_010247 | circRNA | down | -1.4119 | 0.0219 |
| hsacirc_002967 | circRNA | down | -1.2915 | 0.0246 |
| hsacirc_032290 | circRNA | down | -1.2768 | 0.0207 |
| hsacirc_052668 | circRNA | down | -1.0953 | 0.0411 |
| hsacirc_034052 | circRNA | down | -1.0870 | 0.0437 |
| KCNJ12 | mRNA | up | 4.3817 | 0.0094 |
| SHE | mRNA | up | 2.7372 | 0.0226 |
| GCOM1 | mRNA | up | 2.4237 | 0.0076 |
| NRCAM | mRNA | up | 2.3317 | 0.0088 |
| MYZAP | mRNA | up | 2.2955 | 0.0354 |
| TMEM178B | mRNA | up | 1.9621 | 0.0083 |
| SEMA5A | mRNA | up | 1.8651 | 0.0239 |
| VSTM4 | mRNA | up | 1.8532 | 0.0334 |
| MYOM2 | mRNA | up | 1.8373 | 0.0421 |
| MYL4 | mRNA | up | 1.8347 | 0.0342 |
| CA6 | mRNA | up | 1.7315 | 0.0412 |
| FBXO43 | mRNA | up | 1.7018 | 0.0395 |
| FREM1 | mRNA | up | 1.6184 | 0.0472 |
| TKTL1 | mRNA | up | 1.5075 | 0.0456 |
| NPIPB6 | mRNA | up | 1.4701 | 0.0414 |
| C8orf88 | mRNA | up | 1.3576 | 0.0367 |
| HMCN1 | mRNA | up | 1.3380 | 0.0249 |
| NTSR1 | mRNA | up | 1.2407 | 0.0439 |
| WDR63 | mRNA | up | 1.2318 | 0.0270 |
| CMTM8 | mRNA | up | 1.1828 | 0.0109 |
| CRLF2 | mRNA | up | 1.0764 | 0.0234 |
| GDF15 | mRNA | down | -∞ | 0.0408 |
| RNF17 | mRNA | down | -5.0064 | 0.0309 |
| HYDIN | mRNA | down | -4.1067 | 0.0282 |
| HLA-DQA1 | mRNA | down | -2.7341 | 0.0003 |
| RSPO4 | mRNA | down | -2.6673 | 0.0387 |
| MUC19 | mRNA | down | -2.5481 | 0.0435 |
| TCF7L1 | mRNA | down | -2.3603 | 0.0440 |
| COL26A1 | mRNA | down | -2.1634 | 0.0008 |
| HOXA10 | mRNA | down | -2.1264 | 0.0019 |
| C7orf61 | mRNA | down | -2.1190 | 0.0122 |
| PPARGC1A | mRNA | down | -1.8481 | 0.0426 |
| HLA-DQB1 | mRNA | down | -1.8316 | 0.0387 |
| ITGA1 | mRNA | down | -1.7713 | 0.0065 |
| VWDE | mRNA | down | -1.7559 | 0.0000 |
| TRIM64B | mRNA | down | -1.7547 | 0.0120 |
| IQSEC3 | mRNA | down | -1.7488 | 0.0150 |
| UTS2B | mRNA | down | -1.7468 | 0.0143 |
| SCN4A | mRNA | down | -1.7436 | 0.0297 |
| CORO2B | mRNA | down | -1.7258 | 0.0123 |
| SHISA4 | mRNA | down | -1.6991 | 0.0273 |
| CLEC4F | mRNA | down | -1.6561 | 0.0368 |
| OLFM4 | mRNA | down | -1.6417 | 0.0237 |
| ZNF208 | mRNA | down | -1.5185 | 0.0173 |
| ZNF135 | mRNA | down | -1.5028 | 0.0099 |
| DDTL | mRNA | down | -1.5014 | 0.0027 |
| DDX11 | mRNA | down | -1.4518 | 0.0002 |
| CD68 | mRNA | down | -1.4257 | 0.0457 |
| USP50 | mRNA | down | -1.3196 | 0.0489 |
| RAB40AL | mRNA | down | -1.3111 | 0.0442 |
| TMEM255A | mRNA | down | -1.2566 | 0.0085 |
| CYP2F1 | mRNA | down | -1.2358 | 0.0418 |
| UBQLNL | mRNA | down | -1.2004 | 0.0075 |
| MYO16 | mRNA | down | -1.1873 | 0.0245 |
| B4GALNT3 | mRNA | down | -1.1104 | 0.0351 |
| GEM | mRNA | down | -1.0702 | 0.0494 |
| ZNF667 | mRNA | down | -1.0643 | 0.0226 |
| KIAA1324L | mRNA | down | -1.0453 | 0.0334 |

## **Supplementary Table 1**

**Supplementary Table 2. Relationship between negative regulation of circRNA, miRNA, and mRNA in the ceRNA network.**

| **CircRNAs** | **Regulation** | **miRNAs** | **Regulation** | **mRNAs** | **Regulation** |
| --- | --- | --- | --- | --- | --- |
| hsacirc_038403 | Down | hsa-miR-1277-5p | up | PPARGC1A | Down |
| hsacirc_047820 |  |  |  |  |  |
| hsacirc_029293 |  |  |  |  |  |
| hsacirc_014877 |  |  |  |  |  |
| hsacirc_018621 |  |  |  |  |  |
| hsacirc_047424 |  |  |  |  |  |
| hsacirc_049089 |  |  |  |  |  |
| hsacirc_028814 |  |  |  |  |  |
|  |  |  |  |  |  |
| hsacirc_052668 | Down | hsa-miR-1291 | up | IQSEC3 | Down |
| hsacirc_049089 |  |  |  |  |  |
| hsacirc_024833 |  |  |  |  |  |
| hsacirc_038403 |  |  |  |  |  |
|  |  |  |  |  |  |
| hsacirc_022992 | Down | hsa-miR-144-3p | up | HOXA10 | Down |
| hsacirc_038403 |  |  |  |  |  |
| hsacirc_016302 |  |  |  |  |  |
|  |  |  |  |  |  |
| hsacirc_024833 | Down | hsa-miR-149-3p | up | IQSEC3 | Down |
| hsacirc_052668 |  |  |  |  |  |
| hsacirc_012036 |  |  |  |  |  |
| hsacirc_019528 |  |  |  |  |  |
| hsacirc_047424 |  |  |  |  |  |
| hsacirc_038403 |  |  |  |  |  |
|  |  |  |  |  |  |
| hsacirc_018621 | Down | hsa-miR-15a-5p | up | HOXA10 | Down |
| hsacirc_029293 |  |  |  |  |  |
| hsacirc_002822 |  |  |  |  |  |
| hsacirc_038403 |  |  |  |  |  |
| hsacirc_029293 |  |  |  |  |  |
| hsacirc_018621 |  |  |  |  |  |
| hsacirc_038403 |  |  |  |  |  |
| hsacirc_002822 |  |  |  |  |  |
|  |  |  |  |  |  |
| hsacirc_002822 | Down | hsa-miR-16-5p | up | HOXA10、TMEM255A | Down |
| hsacirc_038403 |  |  |  |  |  |
| hsacirc_018621 |  |  |  |  |  |
| hsacirc_029293 |  |  |  |  |  |
| hsacirc_002822 |  |  |  |  |  |
| hsacirc_038403 |  |  |  |  |  |
| hsacirc_018621 |  |  |  |  |  |
| hsacirc_029293 |  |  |  |  |  |
|  |  |  |  |  |  |
| hsacirc_014877 | Down | hsa-miR-190a-3p | up | PPARGC1A | Down |
| hsacirc_033840 |  |  |  |  |  |
| hsacirc_047424 |  |  |  |  |  |
| hsacirc_038146 |  |  |  |  |  |
| hsacirc_012036 |  |  |  |  |  |
| hsacirc_022992 |  |  |  |  |  |
| hsacirc_028814 |  |  |  |  |  |
| hsacirc_038403 |  |  |  |  |  |
| hsacirc_049089 |  |  |  |  |  |
|  |  |  |  |  |  |
| hsacirc_038403 | Down | hsa-miR-195-5p | up | HOXA10 | Down |
| hsacirc_002822 |  |  |  |  |  |
| hsacirc_018621 |  |  |  |  |  |
| hsacirc_029293 |  |  |  |  |  |
|  |  |  |  |  |  |
| hsacirc_019015 | Down | hsa-miR-23a-3p | up | PPARGC1A | Down |
| hsacirc_011466 |  |  |  |  |  |
| hsacirc_038403 |  |  |  |  |  |
| hsacirc_012997 |  |  |  |  |  |
| hsacirc_049089 |  |  |  |  |  |
| hsacirc_024833 |  |  |  |  |  |
| hsacirc_022992 |  |  |  |  |  |
|  |  |  |  |  |  |
| hsacirc_050327 | Down | hsa-miR-27a-3p | up | HOXA10 | Down |
| hsacirc_050343 |  |  |  |  |  |
| hsacirc_034052 |  |  |  |  |  |
| hsacirc_038403 |  |  |  |  |  |
| hsacirc_010247 |  |  |  |  |  |
| hsacirc_010247 |  |  |  |  |  |
| hsacirc_050343 |  |  |  |  |  |
| hsacirc_034052 |  |  |  |  |  |
| hsacirc_038403 |  |  |  |  |  |
| hsacirc_050327 |  |  |  |  |  |
|  |  |  |  |  |  |
| hsacirc_052668 | Down | hsa-miR-29a-5p | up | HOXA10 | Down |
| hsacirc_014877 |  |  |  |  |  |
| hsacirc_049089 |  |  |  |  |  |
| hsacirc_032801 |  |  |  |  |  |
| hsacirc_012036 |  |  |  |  |  |
| hsacirc_038403 |  |  |  |  |  |
|  |  |  |  |  |  |
| hsacirc_018621 | Down | hsa-miR-30a-3p | up | KIAA1324L | Down |
|  |  |  |  |  |  |
| hsacirc_019528 | Down | hsa-miR-30a-3p | up | KIAA1324L | Down |
| hsacirc_040007 |  |  |  |  |  |
| hsacirc_011466 |  |  |  |  |  |
| hsacirc_040187 |  |  |  |  |  |
| hsacirc_023800 |  |  |  |  |  |
| hsacirc_049089 |  |  |  |  |  |
| hsacirc_026711 |  |  |  |  |  |
| hsacirc_014877 |  |  |  |  |  |
| hsacirc_033840 |  |  |  |  |  |
| hsacirc_038403 |  |  |  |  |  |
| hsacirc_047424 |  |  |  |  |  |
| hsacirc_010247 |  |  |  |  |  |
|  |  |  |  |  |  |
| hsacirc_018621 | Down | hsa-miR-30b-3p | up | IQSEC3 | Down |
| hsacirc_016302 |  |  |  |  |  |
| hsacirc_050363 |  |  |  |  |  |
| hsacirc_038403 |  |  |  |  |  |
| hsacirc_034753 |  |  |  |  |  |
| hsacirc_024833 |  |  |  |  |  |
| hsacirc_050343 |  |  |  |  |  |
| hsacirc_052668 |  |  |  |  |  |
| hsacirc_026970 |  |  |  |  |  |
|  |  |  |  |  |  |
| hsacirc_018621 | Down | hsa-miR-30d-3p | up | KIAA1324L | Down |
| hsacirc_023800 |  |  |  |  |  |
| hsacirc_019528 |  |  |  |  |  |
| hsacirc_038403 |  |  |  |  |  |
| hsacirc_014877 |  |  |  |  |  |
| hsacirc_011466 |  |  |  |  |  |
| hsacirc_040187 |  |  |  |  |  |
| hsacirc_047424 |  |  |  |  |  |
| hsacirc_010247 |  |  |  |  |  |
| hsacirc_049089 |  |  |  |  |  |
| hsacirc_026711 |  |  |  |  |  |
| hsacirc_033840 |  |  |  |  |  |
| hsacirc_040007 |  |  |  |  |  |
|  |  |  |  |  |  |
| hsacirc_023800 | Down | hsa-miR-30e-3p | up | KIAA1324L | Down |
| hsacirc_019528 |  |  |  |  |  |
| hsacirc_018621 |  |  |  |  |  |
| hsacirc_014877 |  |  |  |  |  |
| hsacirc_010247 |  |  |  |  |  |
| hsacirc_038403 |  |  |  |  |  |
| hsacirc_040187 |  |  |  |  |  |
| hsacirc_011466 |  |  |  |  |  |
| hsacirc_047424 |  |  |  |  |  |
| hsacirc_040007 |  |  |  |  |  |
| hsacirc_049089 |  |  |  |  |  |
| hsacirc_033840 |  |  |  |  |  |
| hsacirc_026711 |  |  |  |  |  |
|  |  |  |  |  |  |
| hsacirc_038403 | Down | hsa-miR-32-3p | up | PPARGC1A | Down |
| hsacirc_049089 |  |  |  |  |  |
| hsacirc_047424 |  |  |  |  |  |
| hsacirc_050343 |  |  |  |  |  |
|  |  |  |  |  |  |
| hsacirc_038403 | Down | hsa-miR-424-5p | up | HOXA10 | Down |
| hsacirc_029293 |  |  |  |  |  |
| hsacirc_018621 |  |  |  |  |  |
| hsacirc_002822 |  |  |  |  |  |
|  |  |  |  |  |  |
| hsacirc_038403 | Down | hsa-miR-486-5p | up | OLFM4 | Down |
| hsacirc_033508 |  |  |  |  |  |
| hsacirc_018621 |  |  |  |  |  |
| hsacirc_037187 |  |  |  |  |  |
| hsacirc_049089 |  |  |  |  |  |
| hsacirc_012677 |  |  |  |  |  |
|  |  |  |  |  |  |
| hsacirc_016302 | Down | hsa-miR-494-3p | up | HOXA10 | Down |
| hsacirc_038403 |  |  |  |  |  |
| hsacirc_034052 |  |  |  |  |  |
| hsacirc_014877 |  |  |  |  |  |
| hsacirc_018621 |  |  |  |  |  |
|  |  |  |  |  |  |
| hsacirc_002822 | Down | hsa-miR-497-5p | up | HOXA10 | Down |
| hsacirc_038403 |  |  |  |  |  |
| hsacirc_018621 |  |  |  |  |  |
| hsacirc_029293 |  |  |  |  |  |
|  |  |  |  |  |  |
| hsacirc_016302 | Down | hsa-miR-584-5p | up | ZNF208 | Down |
| hsacirc_014877 |  |  |  |  |  |
| hsacirc_038403 |  |  |  |  |  |
|  |  |  |  |  |  |
| hsacirc_049089 | Down | hsa-miR-603 | up | PPARGC1A | Down |
| hsacirc_038403 |  |  |  |  |  |
| hsacirc_012677 |  |  |  |  |  |
| hsacirc_050327 |  |  |  |  |  |
| hsacirc_052668 |  |  |  |  |  |
|  |  |  |  |  |  |
| hsacirc_052668 | Down | hsa-miR-607 | up | GEM | Down |
| hsacirc_049089 |  |  |  |  |  |
| hsacirc_026711 |  |  |  |  |  |
| hsacirc_016302 |  |  |  |  |  |
| hsacirc_020723 |  |  |  |  |  |
| hsacirc_038403 |  |  |  |  |  |
| hsacirc_026970 |  |  |  |  |  |
| hsacirc_014877 |  |  |  |  |  |
| hsacirc_029293 |  |  |  |  |  |
|  |  |  |  |  |  |
| hsacirc_026970 | Down | hsa-miR-625-5p | up | IQSEC3 | Down |
| hsacirc_038403 |  |  |  |  |  |
| hsacirc_050327 |  |  |  |  |  |
| hsacirc_047820 |  |  |  |  |  |
| hsacirc_047424 |  |  |  |  |  |
| hsacirc_018621 |  |  |  |  |  |
|  |  |  |  |  |  |
| hsacirc_038403 | Down | hsa-miR-651-3p | up | HOXA10 | Down |
| hsacirc_026970 |  |  |  |  |  |
| hsacirc_012997 |  |  |  |  |  |
| hsacirc_052668 |  |  |  |  |  |
| hsacirc_023800 |  |  |  |  |  |
| hsacirc_028814 |  |  |  |  |  |
| hsacirc_018621 |  |  |  |  |  |
| hsacirc_049089 |  |  |  |  |  |
|  |  |  |  |  |  |
| hsacirc_038403 | Down | hsa-miR-765 | up | IQSEC3 | Down |
| hsacirc_047491 |  |  |  |  |  |
| hsacirc_037187 |  |  |  |  |  |
|  |  |  |  |  |  |
| hsacirc_034395 | Up | hsa-miR-1206 | down | VSTM4 | Up |
| hsacirc_031080 |  |  |  |  |  |
| hsacirc_035125 |  |  |  |  |  |
| hsacirc_038268 |  |  |  |  |  |
|  |  |  |  |  |  |
| hsacirc_034395 | Up | hsa-miR-1236-3p | down | TMEM178B | Up |
| hsacirc_044577 |  |  |  |  |  |
| hsacirc_011084 |  |  |  |  |  |
| hsacirc_040719 |  |  |  |  |  |
| hsacirc_017357 |  |  |  |  |  |
| hsacirc_008119 |  |  |  |  |  |
| hsacirc_014546 |  |  |  |  |  |
|  |  |  |  |  |  |
| hsacirc_008119 | Up | hsa-miR-124-3p | down | SHE | Up |
|  |  |  |  |  |  |
| hsacirc_031577 | Up | hsa-miR-1299 | down | HMCN1 | Up |
| hsacirc_044577 |  |  |  |  |  |
| hsacirc_035125 |  |  |  |  |  |
| hsacirc_020603 |  |  |  |  |  |
|  |  |  |  |  |  |
| hsacirc_019562 | Up | hsa-miR-190a-3p | down | TMEM178B | Up |
|  |  |  |  |  |  |
| hsacirc_032857 | Up | hsa-miR-212-5p | down | TMEM178B | Up |
| hsacirc_035125 |  |  |  |  |  |
| hsacirc_008119 |  |  |  |  |  |
| hsacirc_034395 |  |  |  |  |  |
|  |  |  |  |  |  |
| hsacirc_010451 | Up | hsa-miR-218-5p | down | SEMA5A | Up |
| hsacirc_028505 |  |  |  |  |  |
| hsacirc_019901 |  |  |  |  |  |
|  |  |  |  |  |  |
| hsacirc_044577 | Up | hsa-miR-508-5p | down | SHE | Up |
| hsacirc_020603 |  |  |  |  |  |
| hsacirc_035125 |  |  |  |  |  |
| hsacirc_032857 |  |  |  |  |  |
|  |  |  |  |  |  |
| hsacirc_038504 | Up | hsa-miR-548c-3p | down | SEMA5A | Up |
| hsacirc_034395 |  |  |  |  |  |
| hsacirc_000273 |  |  |  |  |  |
| hsacirc_032857 |  |  |  |  |  |
| hsacirc_044530 |  |  |  |  |  |
|  |  |  |  |  |  |
| hsacirc_041434 | Up | hsa-miR-656-3p | down | TMEM178B | Up |
| hsacirc_028505 |  |  |  |  |  |
| hsacirc_043227 |  |  |  |  |  |
| hsacirc_044577 |  |  |  |  |  |
| hsacirc_003470 |  |  |  |  |  |
| hsacirc_011084 |  |  |  |  |  |
