# Supplementary figures and images for: Construction of a ceRNA network and screening of potential biomarkers and molecular targets in male smokers with chronic obstructive pulmonary disease
Source: Front Genet. 2024 Jun 12;15:1376721. doi: 10.3389/fgene.2024.1376721 (PMC11199688; doi:10.3389/fgene.2024.1376721)

A

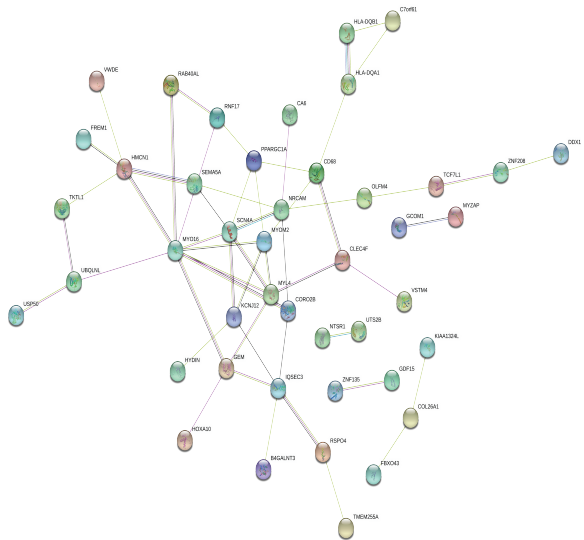

# B

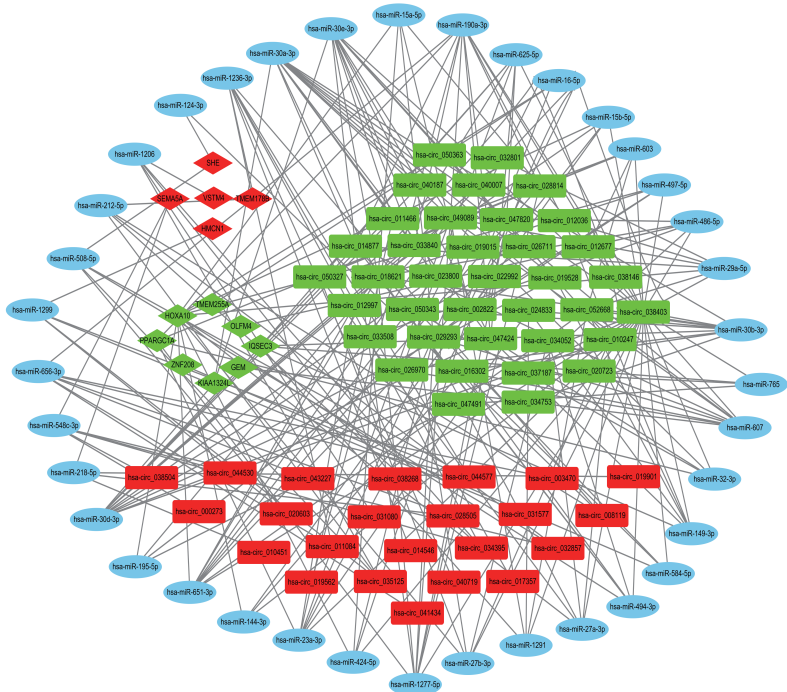

Supplement: Supplementary file 6 [file DataSheet1.PDF]
